# ADD domain added new binding partners for the nuclear hub protein ATRX: ADD domain added new binding partners for ATRX

**DOI:** 10.3724/abbs.2025140

**Published:** 2025-09-08

**Authors:** Yan Chen, Yang Luo, Jielin Sun, Shouhua Wang, Bingbing Wan

**Affiliations:** 1 Key Laboratory of Systems Biomedicine (Ministry of Education) Shanghai Center for Systems Biomedicine Shanghai Jiao Tong University Shanghai 200240 China; 2 Shanghai Key Laboratory of Gene Editing and Cell-based Immunotherapy for Hematological Diseases Shanghai Jiao Tong University Shanghai 200240 China; 3 Institute of Translational Medicine Shanghai Jiao Tong University Shanghai 200240 China; 4 Department of General Surgery Xinhua Hospital Shanghai Jiao Tong University School of Medicine Shanghai 200092 China; 5 National Center for International Research on Systems Biomedicine (Ministry of Science and Technology) Shanghai Jiao Tong University Shanghai 200240 China.

ATRX is a large, multi-domain nuclear protein that functions as a crucial ATP-dependent chromatin remodeler, transcriptional regulator, and guardian of telomeric and genomic integrity [1]. As a member of the SWI/SNF family of chromatin remodeling proteins, a primary and well-defined function of ATRX is to facilitate the replication-independent deposition of the histone variant H3.3 at specific genomic loci, predominantly repetitive sequences such as telomeres, pericentromeric heterochromatin, and ribosomal DNA (rDNA). Mutations in the ATRX gene are associated with a severe X-linked neurodevelopmental disorder and alpha-thalassemia. Moreover, ATRX dysfunction can lead to genomic instability, contributing to the development and progression of various cancers, including gliomas and pancreatic neuroendocrine tumors (PanNETs) [2].

ATRX orchestrates chromatin dynamics through its modular domains. Its N-terminal ADD domain and a PxVxL-like motif recognize histone H3K9me3 and interact with the heterochromatin-binding protein HP1α, respectively. Collaborating with the histone chaperone DAXX, ATRX then utilizes its C-terminal ATPase/Helicase domain to provide the energy needed to remodel chromatin and deposit the histone variant H3.3 into repetitive DNA regions ( Figure 1A) [3]. The Chen lab and other two groups previously elucidated the minimal elements for DAXX interaction, demonstrating that a short 1260‒1289 residues motif (DAXX-binding motif, DBM) of ATRX is solely responsible for ATRX-DAXX heterodimer formation [ 4– 6] . Beyond its role in chromatin remodeling, ATRX employs its RBR (RNA-binding region) to engage the telomeric long non-coding RNA (lncRNA) TERRA, thereby regulating TERRA-mediated R-loops and telomeric G-quadruplex (G4) structures. Interestingly, this same RBR also binds the muscle-specific lncRNA ChRO1 to coordinate constitutive heterochromatin reorganization and regulate cell differentiation. Notably, nearly half of the disease-causing mutations in ATRX [7], leading to a severe neurodevelopmental disorder, are clustered within the ADD domain, highlighting this relatively small domain’s critical functional importance and warranting more intense investigation.

**Figure 1:**
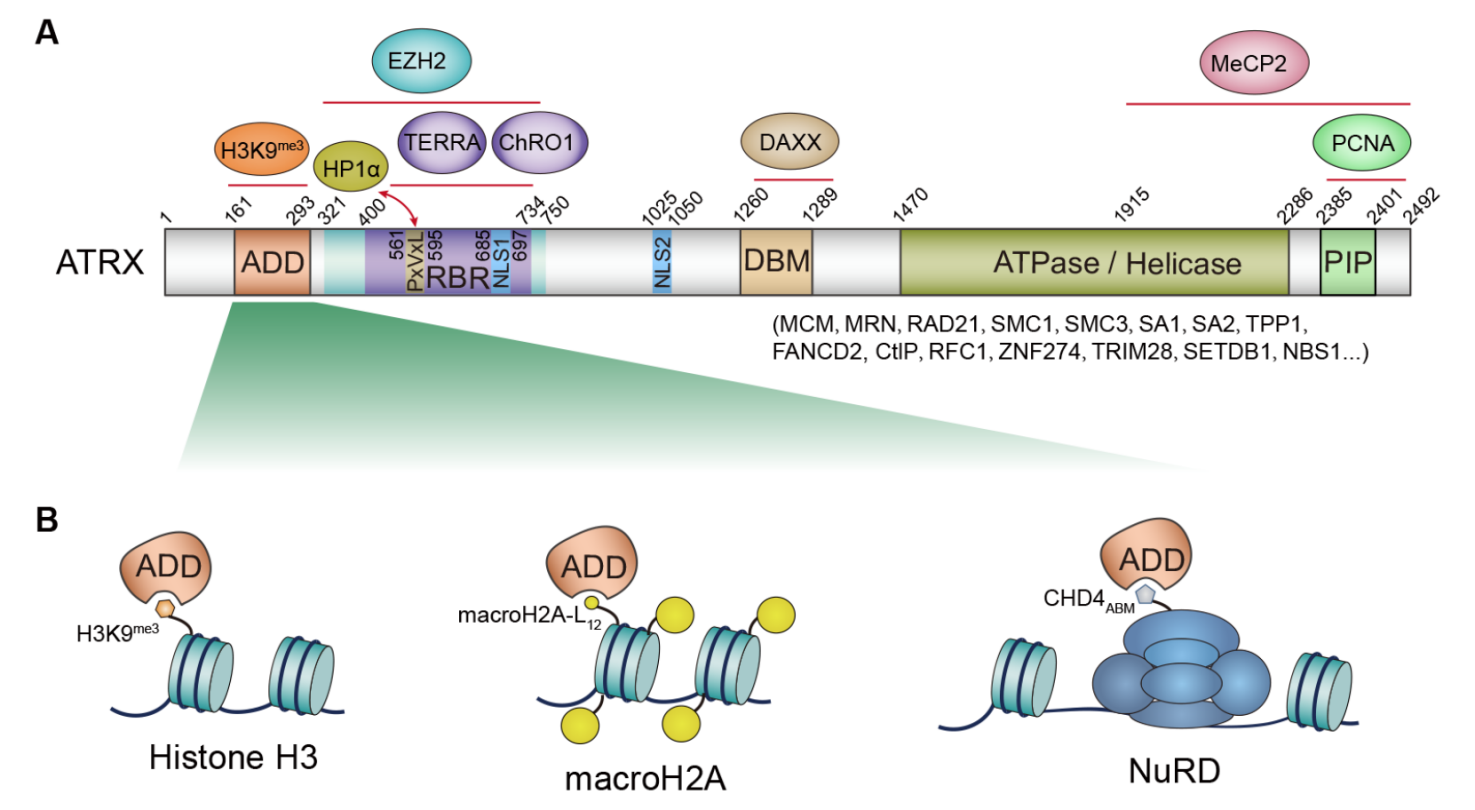
Figure 1 The multifaceted protein ATRX orchestrates a complex regulatory network to modulate epigenetic landscapes and safeguard genomic stability (A) Schematic diagram of full-length ATRX (282 kDa) depicting key protein interaction domains. The ADD domain serves as a binding site for H3K9me3, and the PxVxL motif facilitates interaction with HP1α. The RBR (RNA-binding region) engages with TERRA and ChRO1 lncRNAs, with an overlapping area also binding EZH2. The DBM (DAXX-binding motif) interacts with DAXX, and the C-terminal conserved hydrophobic PIP (PCNA-interacting protein) motif likely mediates PCNA interaction. Additionally, a substantial C-terminal segment (residues 1915‒2492), encompassing a portion of the ATPase/Helicase domain, interacts with MeCP2. (B) The versatile ADD domain coordinate diverse protein interactions. Beyond its established role in binding histone H3K9me3, new research by Yan et al. [12] in this issue significantly broadens the ADD domain’s functional interaction network. Their structural and AP-MS analyses identify the histone variant macroH2A and CHD4, a component of the chromatin remodeling NuRD complex, as new binding partners.

## ADD Domain Functions in Chromatin-Binding Protein ATRX and DNMTs

The ADD domain is a compact, globular domain, typically around 140 residues in length. Its most well-understood function is its ability to recognize and bind to specific post-translational modifications on the N-terminal tail of histone H3. This domain’s unique three-dimensional structure is characterized by the intricate packing of three distinct modules: an N-terminal GATA-like C2C2-type zinc finger, a central C4C4-type PHD zinc finger, and a C-terminal single α-helix [8]. Beyond histone recognition, the DNMT3A ADD domain is known to bind to unmodified or post-translationally modified peptides from proteins such as HDAC1, SETDB1, histone H4R3me2, and EZH2 [9]. This multifaceted binding property resembles the more evolutionarily conserved chromatin-binding BRCT (BRCA1 C-terminal) domain [ 10, 11] , which recognizes phosphorylated histone H2A and various unmodified DNA repair proteins from yeast to humans. Intriguingly, whether ATRX also utilizes its ADD domain to engage a distinct set of proteins, similar to DNMT3A, is still enigmatic until Yan *et al*. [12] reported in this issue, through structural analysis, that the histone variant macroH2A binds the ATRX _ADD_ domain.

To investigate the macroH2A-binding region of ATRX, Yan *et al*. first utilized a GST-pulldown assay with purified macroH2A-H2B heterodimer and truncated ATRX proteins. This approach successfully narrowed the interaction site from the previously reported 1‒841 amino acid region [13] to the more precise N-terminal ADD domain (161‒292 residues). Their analysis further demonstrated that the compact N-terminal three-helix bundle-shaped histone fold (HF) domain of macroH2A-H2B, rather than its macro domain, mediates this interaction. Notably, while confirming the previously reported interaction between macroH2A1-H2B and ATRX [13], the study extended these findings by identifying a direct interaction between ATRX and its structural homolog macroH2A2-H2B ( Figure 1A).

## Molecular Basis of ATRX _ADD_ Domain Recognition of macroH2A-H2B

To delineate the precise interaction, Yan *et al*. [12] leveraged AlphaFold2-multimer to predict the macroH2A1-H2B-ATRX complex structure. The high prediction local distance difference test (pLDDT) values and low predicted aligned error (PAE) rates indicated high reliability of the predicted model. Their analysis revealed that the interaction between ATRX and macroH2A1-H2B is primarily driven by electrostatic forces. Specifically, a negatively charged loop (amino acids 207‒225) connecting the GATA-like and PHD zinc finger of ATRX, rich in aspartic (D) and glutamic (E) residues, docks onto a positively charged surface of macroH2A1 _HF_ L _12_ (linking α-helix-1 and α-helix-2 of macroH2A1 _HF_). They pinpointed that ATRX residues Y203, D207, D208, E218 and macroH2A1 residue R40 play the most critical roles in this engagement. Furthermore, the guanidinium group of macroH2A1 _HF_ L _12_ R40 forms a cation–π interaction with the phenol ring of ATRX Y203, additionally strengthening the overall electrostatic and hydrogen bonding network between ATRX and macroH2A.

To dissect the molecular basis by which ATRX distinguishes between canonical histone H2A and macroH2A, Yan *et al*. [12] performed a comparative analysis of their respective nucleosome core particle (NCP) structures. They identified that the preceding residues _36_HPKY _39_ of macroH2A1 L _12_ residue R40 adopts a configuration distinct from the corresponding _39_NYAE _42_ segment of canonical H2A (containing R43). This unique conformation of the macroH2A segment facilitates closer contacts with the ATRX _ADD_ domain. Strikingly, swapping the _39_NYAE _42_ of H2A segment into _36_HPKY _39_ of macroH2A1 L _12_ abrogated ATRX binding, whereas the reverse swap in H2A awarded ATRX binding capacity.

Does the ATRX _ADD_ domain employ a similar mechanism to bind both macroH2A and H3K9me3? Yan and colleagues addressed this question through competitive GST pull-down assays and isothermal titration calorimetry (ITC). Their experiments demonstrated that increasing concentrations of H3K9me3 peptide progressively reduced macroH2A/H2B-ATRX binding. ITC measurements revealed the wild-type H3K9me3 peptide binds ATRX with a dissociation constant (K _d_) of around 4 μM, while Y203A and D/E mutations in ATRX significantly weakened this interaction. Moving forward, it will be crucial to further explore whether macroH2A or H3K9me3 binds ATRX more strongly under both *in vitro* and *in vivo* conditions.

## New ATRX Binding Partners Are Added Through Its ADD Domain

The DNMT3A _ADD_ domain is well-known as a versatile module involved in diverse protein interactions [9]. Intriguingly, the ATRX _ADD_ domain utilizes a similar surface region to bind both H3K9me3 and macroH2A. This similarity raised the possibility that the ATRX ADD domain might interact with other proteins beyond histones. Indeed, Yan and colleagues employed conventional AP-MS to identify numerous new ATRX-interacting proteins. These included several components of the Nucleosome Remodeling and Deacetylase (NuRD) complex, such as MTA1/2, HDAC1/2, RBBP4/7, CHD4, and MBD2/3. Subsequent experiments confirmed these interactions, as Flag-tagged ATRX successfully co-purified endogenous NuRD complex proteins including CHD4, MTA2, and HDAC2. Leveraging AlphaFold3, a short fragment within the N-terminal disordered region of CHD4 (termed CHD4 ADD-binding motif, or CHD4 _ABM_) was predicted to pair with the ATRX ADD domain by forming a β-strand. Interestingly, CHD4 _ABM_ and H3K9me3 share structural commonalities: both adopt very similar β-strand configurations and possess key AR/KTK residues at the N-terminus of their respective peptides. Notably, ITC revealed a K _d_ of approximately 46 μM for the binding of CHD4 _ABM_ to ATRX. This interaction was further validated by ITC experiments using ATRX Y203A and D/E mutations, known to impair its binding.

While the structural and biochemical data presented by Yan *et al*. [12] have substantially advanced our comprehension of the ATRX _ADD_ domain, several critical questions remain to be addressed.

First, despite the strong association between ATRX _ADD_ mutations and human diseases, the key residues identified for H3K9me3, macroH2A, and CHD4 interactions do not align with the most prevalent disease-causing point mutations within the ADD domain [ 7, 14] . This discrepancy strongly suggests that these disease-associated residues may serve as docking sites for other crucial, yet-to-be-discovered, ATRX _ADD_-interacting proteins. Identifying these unknown binding partners could directly illuminate the etiology of ATR-X syndrome and other diseases.

Second, a pertinent question arises regarding the DNMT3A _ADD_ domain: does it, like its ATRX counterpart, utilize a similar competitive binding strategy to interact with its diverse set of functionally distinct partners, including HDAC1, SETDB1, histone H4R3me2, and EZH2?

Finally, the physiological and pathological roles of the newly identified ATRX ADD-CHD4 interaction in cell and mouse models warrant further investigation. Given that the ADD domain generally functions as a molecular “GPS” for ATRX, it is crucial to ascertain whether CHD4 _ABM_ actively recruits ATRX to NuRD-associated chromatin regions, thereby enabling ATR’s ATPase/helicase to remodel local chromatin structure. To fully elucidate these aspects, comprehensive functional assays in various cell and mouse models are essential to evaluate this interaction’s potential involvement in transcriptional regulation, cellular differentiation and development, genomic instability, telomere malfunction, or tumorigenesis.
